# Resolving Nanoscale Heterogeneities in Lead Halide Perovskites Through Low‐Dose Concurrent 4D‐STEM‐EDX Mapping

**DOI:** 10.1002/adma.74382

**Published:** 2026-07-31

**Authors:** Jinseok Ryu, Alexandra A. Sheader, Mohsen Danaie, David G. Hopkinson, Katherine E. MacArthur, Nakita K. Noel, Christopher S. Allen

**Affiliations:** ^1^ electron Physical Science Imaging Centre Diamond Light Source Ltd. Didcot UK; ^2^ Department of Physics University of Oxford Oxford UK; ^3^ Oxford Instruments Materials Analysis High Wycombe UK; ^4^ Department of Materials University of Oxford Oxford UK

**Keywords:** crystal structure, electron beam processing, fabrication, materials science, nanoscopic scale, nanotechnology, photovoltaics, scanning transmission electron microscopy, spectroscopy, transmission electron microscopy

## Abstract

Mixed‐cation lead mixed‐halide perovskites are promising materials for applications in photovoltaics; however, it has been suggested that they exhibit instabilities linked to nanoscale heterogeneity. Directly probing the origins of this heterogeneity requires characterization with nanoscale spatial resolution, making transmission electron microscopy (TEM) an essential tool. However, characterizing these materials is challenging due to their extreme sensitivity to electron irradiation. Here, we develop a low‐dose, concurrent methodology using four‐dimensional scanning transmission electron microscopy (4D‐STEM) and energy‐dispersive x‐ray spectroscopy (EDX) in order to map both the chemical and structural architecture of a (FA_0_._83_Cs_0_._17_)Pb(I_0_._8_Br_0_._2_)_3_ perovskite film without inducing damage. Our correlative analysis reveals a complex mosaic of coexisting crystal structures in this state‐of‐the‐art LHP film. We establish a direct link between local chemical composition and crystal structure, showing that the formation of undesirable, photovoltaically inactive hexagonal polytypes is predominantly driven by local deficiencies in the stabilizing cesium cation. These findings provide crucial insight into one of the fundamental origins of structural instabilities in mixed composition perovskite thin‐films, suggesting that achieving long‐term device performance requires the development of fabrication routes that ensure compositional homogeneity at the nanoscale.

## Graphical abstract

Mixed‐cation lead mixed‐halide perovskites suffer from structural instabilities linked to nanoscale heterogeneity. To probe this non‐destructively, a low‐dose, concurrent 4D‐STEM and EDX methodology has been developed. Examining a (FA_0_._83_Cs_0_._17_)Pb(I_0_._8_Br_0_._2_)_3_ film revealed a complex mosaic of coexisting crystal structures. Crucially, local deficiencies of the stabilizing cesium cation drive the formation of inactive hexagonal polytypes, highlighting the need for nanoscale compositional homogeneity.

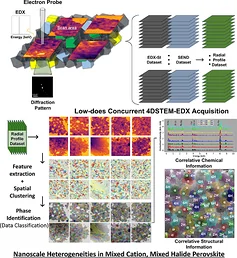

## 1 Introduction

Lead halide perovskites (LHPs) are promising next‐generation photovoltaic materials with certified lab‐scale power conversion efficiencies approaching 27% [1], low manufacturing costs, and excellent bandgap tunability. Formamidinium lead iodide (FAPbI_3_) has attracted considerable interest due to its near‐ideal bandgap for single‐junction photovoltaic devices, and improved thermal stability compared to its methylammonium‐based counterparts [2]. However, its photo‐active, cubic perovskite phase is only thermodynamically metastable at room temperature, readily converting to wide‐bandgap, photoinactive hexagonal polytypes [3]. Alloying with cesium (Cs^+^) and bromide (Br^−^) to form materials such as (FA_0_._83_Cs_0_._17_)Pb(I_0_._8_Br_0_._2_)_3_, has been shown to be an effective strategy for stabilizing the α‐phase perovskite and achieving high‐performance, durable solar cells [3, 4, 5]. While many processing strategies have been shown to enhance material stability and device efficiency, it is probable that LHP films with complex elemental configurations, such as FA^+^/Cs^+^ cations and I^−^/Br^−^ anions, fabricated through standard deposition techniques could exhibit inherent nanoscale heterogeneity [4, 5, 6]. However, the spatial distribution of localized structural and chemical heterogeneities over a large, micrometer‐scale area remains poorly understood.

To understand and ultimately mitigate performance degradation in mixed‐cation, mixed‐halide perovskite materials requires a detailed understanding of nanoscale structural and chemical heterogeneity. Regarding this, transmission electron microscopy (TEM) is a powerful technique that can correlate nanoscale structural and chemical information from the same sample region. However, in the case of lead halide perovskites, its application is severely limited by the material's extreme sensitivity to the electron beam [7, 8]. The high electron dose, which is typically required to obtain accurate sample information using spectroscopic techniques like energy‐dispersive x‐ray spectroscopy (EDX), can induce damage to the materials and cause artifacts, resulting in erroneous data interpretation. While specialized low‐dose techniques such as cryo‐electron microscopy have been successfully applied to probe the crystalline nature of colloids in perovskite precursor inks [9], and advanced scanning TEM (STEM) techniques have revealed nanoscale structural heterogeneity in solid‐state thin‐films [6, 10, 11, 12, 13], the simultaneous acquisition of highly correlated, nanoscale structural and chemical information without inducing degradation remains a considerable challenge.

Here, we develop a concurrent acquisition system for four‐dimensional STEM (4D‐STEM) and EDX. We align microscope optics for scanning electron nanobeam diffraction (SEND), in which a small convergence angle beam with an illumination size on the order of 1 nm is raster scanned across the sample. Diffraction patterns at each beam position are recorded using a high‐efficiency direct electron detector (DED), allowing us to acquire structural data at electron doses low enough to preserve the pristine state of the material. To segment the SEND data into distinct crystalline phases, we employ non‐negative matrix factorization (NMF), a statistical, data‐driven approach, enabling nanoscale phase classification. While Duran et al. demonstrated simultaneous SEND and EDX acquisition using different hardware configurations, their methodology utilized principal component analysis (PCA) and fuzzy C‐means clustering on beam‐insensitive alloys at relatively high doses [14]. In contrast, our EDX data, acquired under stringent low‐dose conditions, inherently possesses extremely low signal intensities, often yielding zero or one x‐ray count per pixel. Consequently, the individual EDX spectra are insufficient for classification based on their spectral features alone. This underscores the necessity of preemptive phase classification using SEND data, followed by the integration of EDX spectra from regions identified as belonging to the same crystalline phase. This approach enhances the signal‐to‐noise ratio (SNR) of EDX data, providing chemical information to supplement the phase classification.

In this work, to investigate the inherent nanoscale phase heterogeneity in mixed‐cation, mixed‐halide perovskite, we apply our low‐dose concurrent 4D‐STEM and EDX methodology to a stable, high‐quality (FA_0_._83_Cs_0_._17_)Pb(I_0_._8_Br_0_._2_)_3_ film targeted for photovoltaic applications [15]. Indeed, J‐V characterization of a photovoltaic device fabricated in parallel with the thin films studied in this work confirms their high quality, yielding a peak power conversion efficiency of 20.5% (Figure S1). Despite the respectable performance of these films when incorporated into devices, our analysis reveals a highly fragmented mosaic where the desired photoactive α‐phase perovskite is intimately intermixed with various photovoltaically inactive hexagonal polytypes. The correlative chemical mapping provides a clear rationale for this phase heterogeneity: the formation of these hexagonal phases is directly linked to local regions of severe Cs deficiency. This study provides direct, nanoscale evidence that incomplete cation alloying is a primary driver of instability, creating a pervasive network of performance‐limiting phase boundaries even in high‐performance, mixed‐cation perovskite films, and highlights the critical need to develop advanced processing methodologies targeted toward achieving compositional homogeneity. While macroscopic device performance is ultimately hindered by nanoscale heterogeneity, mitigating these effects requires a fundamental understanding of the origin of this heterogeneity. It is well‐established that hexagonal polytypes act as recombination centers and limit device longevity [4, 6, 10]. By demonstrating that the formation of these domains is initially highly localized and driven by nanoscale Cs deficiencies, this low‐dose correlative mapping strategy provides a targeted and interlinked structural‐chemical baseline. Such insights are essential for guiding future processing strategies aimed at eliminating these polytypes, hence maximizing macroscopic device efficiency, representing a critical step toward ensuring long‐term stability in perovskite optoelectronics.

## 2 Results and Discussion

### 2.1 Large‐Sized, Multi‐Modal Data Construction and Classification

Given the well‐known instability of LHPs under electron radiation, we first investigated any beam‐induced structural changes that occur during measurement. Using our concurrent acquisition system (Figure S2), we collected two sets of 10 frames of SEND‐EDX data pairs using two different dwell times per pixel: 1 and 2 ms. These correspond to the electron doses per pixel of 10 and 20 e^−^/Å^2^, respectively. For the 1‐ms dwell time data, we observed no significant changes in the intensities of diffraction peaks after repeated scans (Figure 1a–d). However, doubling the dwell time resulted in significant structural changes in the sample and pronounced amorphization (Figure 1e–h). We identified these structural changes as a change in the ratio of Bragg peaks (Figure 1f), while amorphization was identified by a loss in intensity (Figure 1h). As such, all subsequent SEND‐EDX data pairs were acquired using a 1.0 ms dwell time. Crucially, previous dedicated studies have quantified the critical electron dose required to avoid ion migration and structural phase transitions in these highly sensitive materials, establishing safe exposure limits of < 200 e^−^/Å^2^ [11, 12]. Furthermore, state‐of‐the‐art phase‐mapping studies corroborate that operating well below these thresholds successfully prevents beam‐induced structural artifacts [10, 11].

**FIGURE 1:**
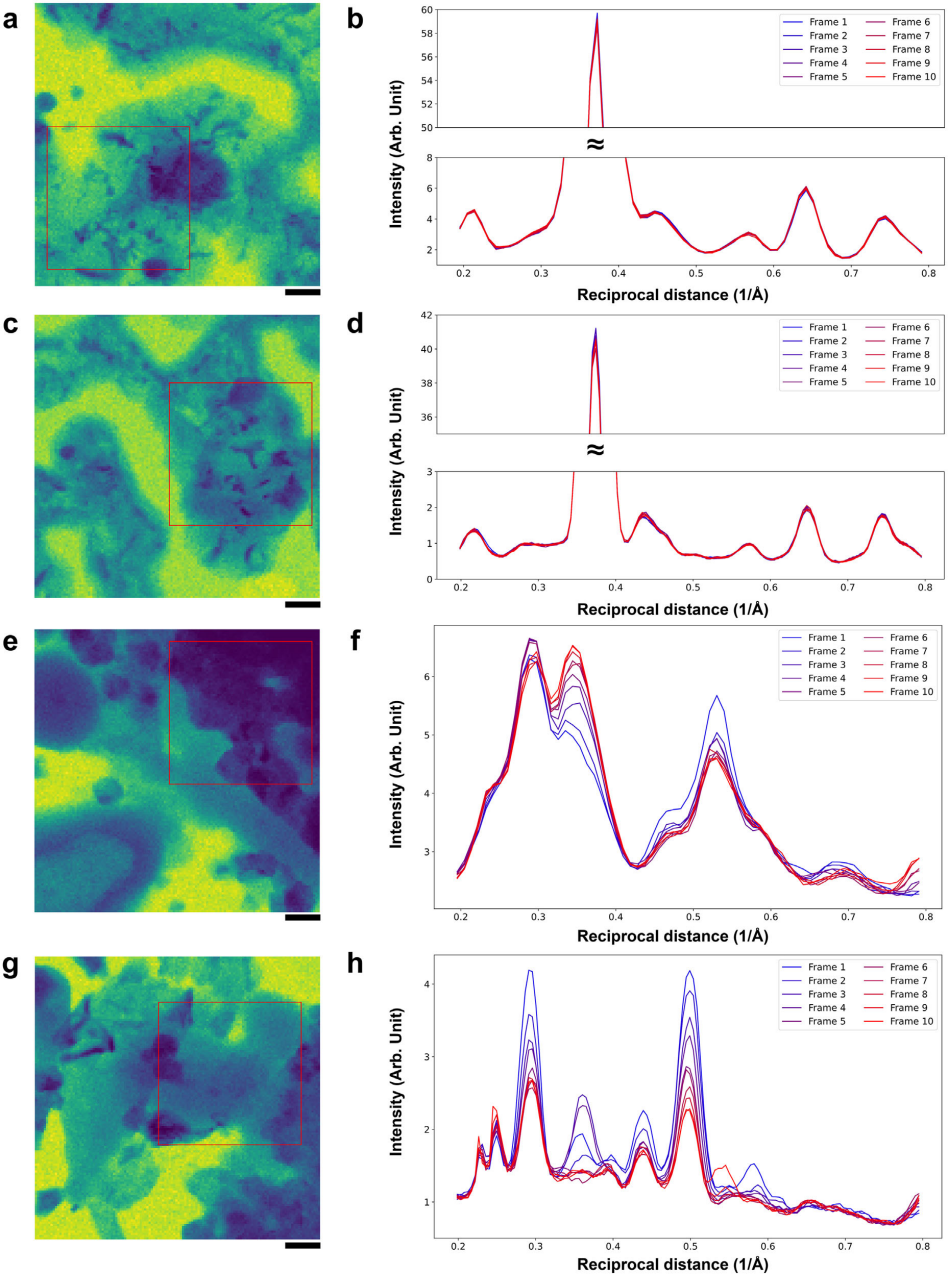
Investigation of Electron Beam‐Induced Degradation in Lead Halide Perovskite Films. (a, c, e, g) Virtual bright‐field (vBF) images of scanning electron nanodiffraction (SEND) data with ten multiple scans and different dwell times per pixel: 1 ms (a, c) and 2 ms (e‐g). Only the vBF images of the first frame SEND data are presented. (b, d, f, h) Their corresponding radial mean profiles (RMPs) Each summed RMP is taken from the red‐bordered regions within the vBF images in the same row. Scale bars = 100 nm.

We recorded 100 SEND‐EDX datasets across various regions of the sample, collectively covering a total area of 63.5 µm^2^ (Figure S3). The median distance between adjacent acquisition locations was 9.6 µm (Figure S4); this sampling strategy was specifically designed to avoid localized biases and capture a statistically representative overview of the film's heterogeneity. Furthermore, we aim to include a broad range of morphologically distinct regions of interest (ROIs), encompassing areas in which the perovskite is deposited both on the carbon support film and suspended over free space, guided by annular dark‐field (ADF) STEM imaging (Figure S5). The electron beam was blanked when moving the sample to ensure that no damage to the sample had occurred before measurements were taken. As the inelastic scattering cross‐section is relatively low and EDX detection efficiency is poor (due to both adsorption in the sample and detection geometry), the number of EDX counts in each pixel of the combined datasets is, on average, close to one. In contrast, the cross section for coherent elastic scattering, which forms diffraction patterns, is significantly higher than the cross section for the specific inelastic interactions that generate characteristic x‐rays for EDX analysis [16]. The detection efficiency of DEDs is also very high, resulting in information‐rich diffraction patterns from each pixel. Therefore, we exploited the high information content in diffraction space to segment our combined SEND‐EDX data into regions corresponding to particular crystallographic phases of the sample.

In the initial classification of the SEND datasets, NMF, a dimensionality reduction method, was employed (see Experimental Section for details) [17]. NMF has been shown to be an effective technique for feature extraction from large datasets such as 4D‐STEM or electron energy loss spectral image data [18, 19, 20, 21, 22, 23, 24, 25, 26, 27]. Here we first reduced each two‐dimensional (2D) diffraction pattern to 1D data by calculating the radial variance profile (RVP) of each pattern prior to NMF decomposition. We note that the amorphous carbon (a‐carbon) support film on the TEM grid also contributes to the diffraction patterns, which makes it difficult to accurately detect diffraction spots. The transformation of 2D diffraction patterns to 1D RVPs enhances the visibility of diffraction features while preserving the identification of reciprocal distances corresponding to Bragg peak positions [28, 29]. Although this data reduction results in the loss of information on crystal symmetry, the reciprocal distance information is retained and is sufficient to identify the distribution of different crystallographic phases.

The result of the NMF decomposition is shown in Figure 2. The RVP datasets depicted in Figure 2a were decomposed into a linear combination of nine loading vectors (LVs), each of which displayed a distinctive variance peak, which represents a strong Bragg reflection at a certain reciprocal distance (Figure 2b). The optimized number of LVs was determined as the point at which the difference in errors between the original and reconstructed data began to plateau (Figure S6). Figure 2c displays the coefficient maps corresponding to nine of the total 100 scan regions, showing the spatial distribution of each LV. A threshold was applied to each coefficient map to include only the top 10% of values, and the resulting regions were clustered using a density‐based clustering algorithm and concave hull method [30, 31] (see Experimental Section for details). A resultant high coefficient mask is shown in Figure 2d, revealing the effective sample areas with a high contribution from each LV (the results of the total datasets can be found in Figures S7 and S8). Interestingly, even though the masking steps were applied to the coefficient maps of each LV independently, very little overlap between individual clusters is observed. This indicates that each cluster contains a high contribution of only one specific LV, likely representing a single‐phase region. Therefore, scan positions that have a strong variance of diffraction intensities at a certain reciprocal distance were classified into one of nine groups based on these features in the RVP datasets through the application of NMF.

**FIGURE 2:**
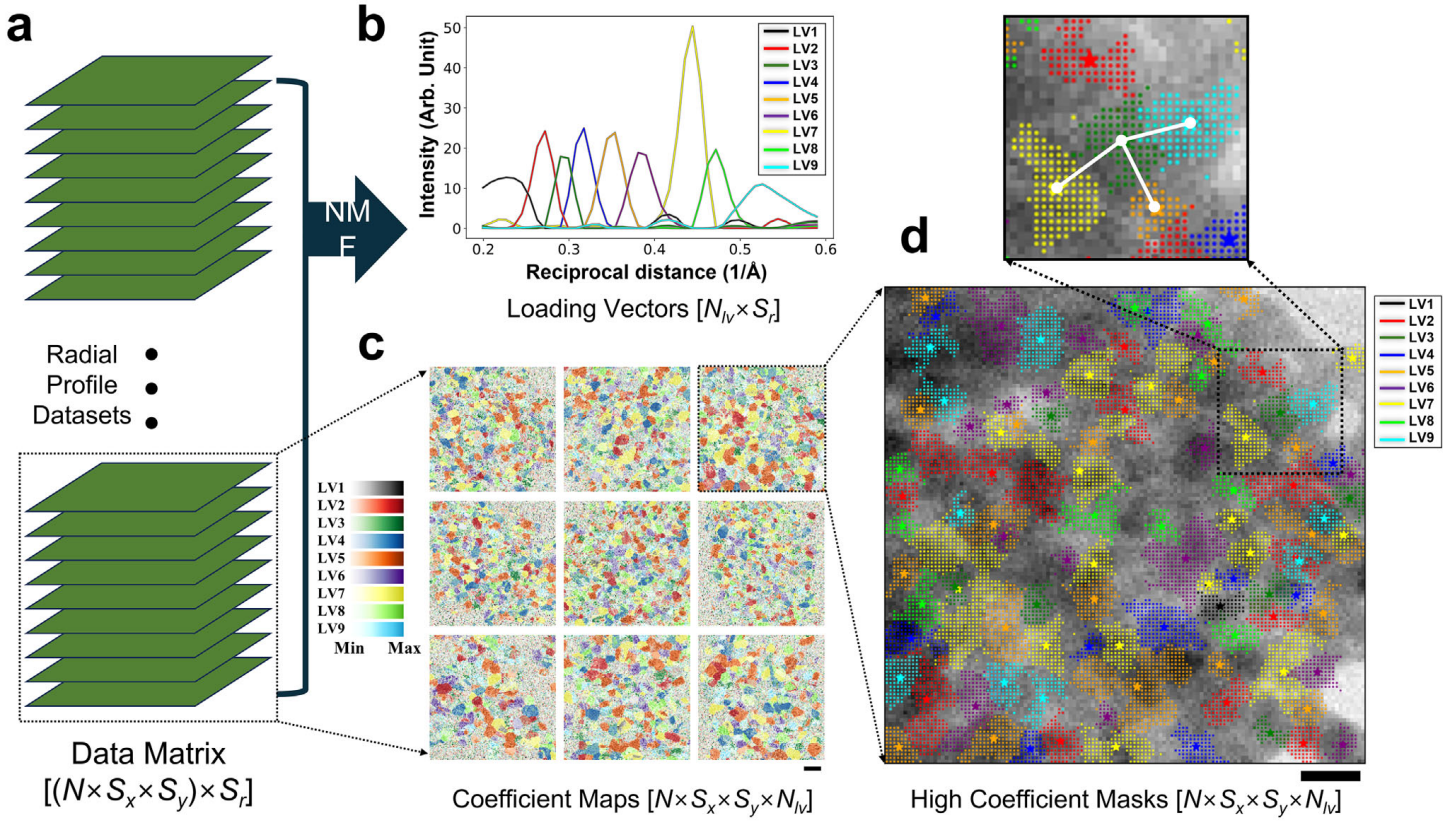
Data classification workflow. (a) One hundred (*N*) radial profile datasets are merged into a data matrix for non‐negative matrix factorization (NMF) decomposition by flattening the scan shape [*S_x_* × *S_y_*]. Only the reciprocal distance range of 0.2–0.6 Å^−1^ is used, which results in 44 reciprocal distance steps (*S_r_*). (b) Nine loading vectors produced by NMF decomposition of the data matrix. (c) NMF coefficient maps; only nine of the 100 scan regions are displayed. (d) Representative example of the high coefficient masks. The colors in the high coefficient mask (d) are consistent with those of the loading vectors, and the mask regions overlaid on a virtual bright‐field image. The star markers in the high coefficient mask represent the centroids of each distinct clustered region. The white lines in the cropped high coefficient mask show the three nearest clusters. The complete sets of coefficient maps and high coefficient masks are provided in the Supplementary Information. Scale bars = 100 nm.

From the SEND data classification by NMF, we are now able to calculate a class‐averaged RVP and EDX spectrum from distinct domains in the sample over all 100 datasets. This approach greatly improved the SNR of both the RVP and EDX data and enables the assignment of structure (see Figure 3 and Figure S9). The class‐averaged RVPs are consistent with the corresponding LVs in terms of their most prominent peaks. Furthermore, Figure 3b shows that the class‐averaged 2D diffraction patterns also have distinctive features between LVs, which suggests that NMF decomposition extracted fundamental feature components from the RVP datasets. By aggregating large numbers of pixels to achieve robust total x‐ray counts (Table S1), prominent peaks corresponding to the characteristic x‐ray emission energies of the constituent elements can be clearly observed in the class‐averaged EDX spectra (Figure S9). Particularly, Figure 3c shows the differences in the intensity of the Cs Lα ionization edge between the class‐averaged EDX spectra. This is not observable in the individual EDX spectra due to the very low counts per spectrum in the raw SEND‐EDX data, as shown in Figure S10.

**FIGURE 3:**
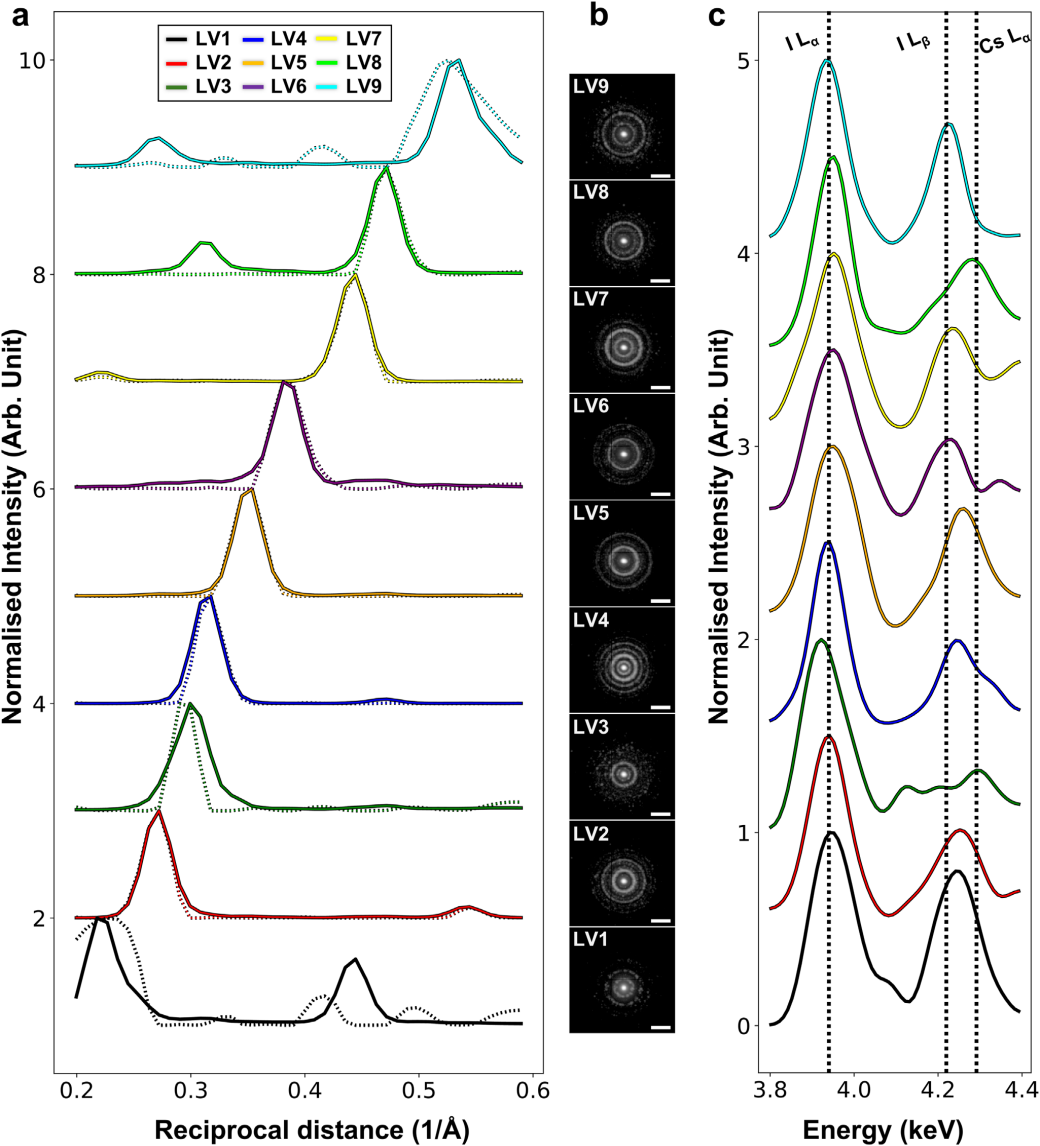
Results of data classification and signal enhancement. (a) Class‐averaged radial variance profiles (RVPs) and their corresponding loading vectors shown with solid and dotted lines, respectively. (b) Class‐averaged raw diffraction patterns. (c) Class‐averaged energy dispersive x‐ray (EDX) spectra presented only in the energy range of 3.8–4.4 keV. Note that Gaussian blurring is applied to the class‐averaged EDX spectra to clearly show the ionization edges of I L_α_, I L_β_, and Cs L_α_. The original class‐averaged EDX spectra are displayed in the Supplementary Information. The edges are displayed with dotted lines with their labels. Scale bars = 0.5 Å^−1.^

### 2.2 Correlative Analysis of Structural and Chemical Heterogeneity

Using bulk x‐ray diffraction techniques, the FA_0.83_Cs_0.17_Pb(I_0.8_Br_0.2_)_3_ perovskite films used in this study appear to be dominated by an α‐phase perovskite with a preferred orientation in the (100) direction (Figure S11). However, it has been reported that FA‐rich LHP films can have a complex mixture of different crystal structures, where the desired photoactive cubic (α) and tetragonal (β) phases coexist with photovoltaically inactive, non‐perovskite hexagonal polytypes (most commonly 2H, 4H, and 6H) [32]. To enable phase identification in the SEND‐EDX datasets, a comprehensive library of candidate phases was prepared for powder x‐ray diffraction (XRD) simulation (Table S2). This library was designed to account for any compositional and structural heterogeneity, including possible degradation products, in the perovskite film [32, 33, 34, 35, 36]. Given that the nominal stoichiometry is heavily weighted toward FAPbI_3_, our candidate library focused on the photoactive (α, β) and photoinactive (2H, 4H, 6H) polytypes associated with the FAPbI_3_ parent structure, although the actual local composition in the film is likely to be mixed FA_x_Cs_1‐x_Pb(I_y_Br_1‐y_)_3_. However, to account for potential local variations in stoichiometry, we also included the end‐member compositions γ‐CsPbI_3_ and α‐FAPbBr_3_. Furthermore, as lead iodide is a key precursor and common decomposition product, the 2H‐ and 3R‐PbI_2_ were also included. To assign the dominant phase to each LV, we compared the class‐averaged RVPs with the simulated XRD patterns.

We then calculated a peak‐position matching score, defined as the sum of the differences between the positions of the peaks in the class‐averaged RVP patterns and expected Bragg reflection positions from XRD simulation of all candidate material phases (see Experimental Section and Supplementary Text for details). Note that the simulated XRD patterns were Gaussian‐blurred to make the full‐width‐at‐half‐maximum (FWHM) of their peaks comparable to that of nanobeam electron diffraction (NBED) patterns, as shown in Figure S12. Furthermore, the method for phase identification depends on the comparison of peak positions; hence, a phase with a relatively high frequency of Bragg reflections, such as γ‐CsPbI_3_, will dominate the best match results without the blurring process. We determined the local maxima of the class‐averaged RVPs and the simulated XRD patterns to obtain their peak positions; at least the positions of the three most prominent peaks are considered necessary for reliable phase identification (Table S3). For each extracted experimental peak, the closest corresponding peak in a given simulated XRD pattern was determined and the absolute difference between the experimental and simulated peak positions measured. The final matching score for a candidate phase is the sum of the absolute positional differences of all peaks (Table S4). For each extracted experimental peak, the closest corresponding peak in a given simulated XRD pattern was determined and the absolute difference between the experimental and simulated peak positions measured. The final matching score for a candidate phase is the sum of the absolute positional differences of all peaks (Table S4). For each LV, the candidate crystal structure yielding the lowest matching score was assigned as the most appropriate phase. Three prominent peaks were successfully extracted and used for structural assignment in all LVs, except for LV8, where only two distinct peaks were identified. In this case the phase with the lowest matching score was the 2H polytype. Although fewer peaks inherently limit the total structural constraints for LV8, the assignment to the 2H polytype remains unambiguous, as its matching score is twice as low as the next closest candidate phase.

In Table 1, the dominant crystal structures identified in each LV are displayed alongside its corresponding EDX quantification. This reveals a complex nanoscale heterogeneity in which regions with the desired photoactive α‐phase perovskite structure are intimately intermixed with photovoltaically inactive hexagonal polytypes. This structural identification is supported by the correlative EDX chemical mapping, which provides a clear explanation for the observed phase heterogeneity. It should be noted that the atomic percentages obtained from EDX quantification may deviate from true, absolute stoichiometry. Based on the integrated x‐ray counts of the stable Pb M‐edge reference, the relative Poisson statistical error for the perovskite and polytype domains is estimated to be between 9% and 19% (Table S1; noting that the largest error of 24% is expectedly observed in the amorphous carbon support film, LV1). However, because this quantification methodology is applied uniformly across all spatially binned regions, these calculated percentages provide a metric for comparing relative chemical variations—such as the observed Cs deficiency—between the different structural phases. The quantification results for the elements of Cs, Pb, I, and Br are displayed in Table 1. The matching result (Table S4) indicates that LV1 is likely to represent the α‐perovskite. However, nearly 80% of the averaged original diffraction patterns taken from the clustered regions of LV1 show no crystallographic Bragg reflections (Figure S13), which suggests that LV1 represents the diffraction features originating from the a‐carbon support film of the TEM grid. As our phase identification analysis reveals that the α‐perovskite structure is the dominant phase in this film, many of the clustered regions of LV1 (a‐carbon) also contain strong diffraction features corresponding to the α‐perovskite phase.

**TABLE 1:** Results from a comprehensive, correlative analysis of nanoscale heterogeneity in the FA_0.83_Cs_0.17_Pb(I_0.8_Br_0.2_)_3_ film.

LV	Identified phase	Most frequent neighboring LV	Area (%)	Cs (at%)	Pb (at%)	Br (at%)	I (at%)
LV1	a‐Carbon	LV1 (a‐Carbon)	15.1	10	22	5	63
LV2	α‐FAPbI_3_	LV8 (2H)	15.4	5	24	15	56
LV3	4H	LV8 (2H)	14.7	0	22	14	64
LV4	α‐FAPbI_3_	LV5 (α‐FAPbI3)	12.2	6	21	13	60
LV5	α‐FAPbI_3_	LV4 (α‐FAPbI3)	12.1	12	17	17	54
LV6	2H	LV2 (α‐FAPbI3)	10.8	3	22	17	58
LV7	α‐FAPbI_3_	LV8 (2H)	6.1	8	20	23	49
LV8	2H	LV7 (α‐FAPbI3)	5.9	9	28	16	48
LV9	6H	LV3 (4H)	7.7	0	18	16	66

Our phase identification analysis shows that the formation of the 4H and 6H polytypes, along with one 2H variant (LV3, LV6, and LV9), is likely linked to local regions of significant Cs deficiency. This is revealed by the low intensities of the Cs Lα ionization edge in the class‐averaged EDX spectra (Figure 3c) and the low atomic percentage of Cs in the EDX quantification result (Table 1). On the other hand, regions with a moderate abundance of Cs (LV2, LV5, and LV7) were identified as the α‐perovskite. The chemical landscape determined from the class‐averaged EDX spectra is consistent with the spatial distribution of the dominant structure determined from the SEND data, which shows the photoinactive hexagonal domains are not clustered but are finely interspersed throughout the α‐perovskite matrix. Our correlative analysis of the SEND‐EDX dataset provides a comprehensive picture of how nanoscale chemical heterogeneity is associated with the complex nanostructure of the film, including the formation of small, undesired domains and a network of structurally distinct phase boundaries.

To further understand the nanoscale heterogeneity in this film, we analyzed the local neighborhood of each phase by investigating the distances between the clustered regions of a source LV and neighboring LV as shown in Figure 2d. By counting the number of the three closest neighboring clustered regions and calculating the percentages of their LVs as displayed in Table S5, we obtained information about spatial correlations between the LVs. This analysis reveals a highly fragmented and intermixed landscape where photoactive and inactive domains are intimately linked. The diagonal values of Table S5 are consistently low, indicating that the domains of each LV are well clustered separately. The exception is LV1, which is likely due to its large contribution from the a‐carbon support film. In contrast, most of the off‐diagonal values are higher than the diagonal values, revealing a network of interfaces between the desired α‐perovskites and the various hexagonal polytypes. For example, the most frequent neighbors of the α‐perovskite variants (LV2 and LV7) are the 2H variant (LV8). In a similar manner, the most frequent neighbors of the 2H variants (LV6 and LV8) are the α‐perovskite variants (LV2 and LV7). Interestingly, our neighboring phase analysis shows that the closest neighbor of the 4H (LV3) is the 2H (LV8), and that 4H (LV3) is the most common neighbor of the 6H phase (LV9). It has been proposed that these structures evolve sequentially, following a thermodynamic crystallization pathway of 2H → 4H → 6H [32]. In this context, it would be anticipated that the 4H and 6H domains should be near the 2H and 4H domains, respectively. Therefore, the spatial correlation revealed in this analysis between the domains of the 2H, 4H, and 6H polytypes supports the proposed crystal evolution pathway.

### 2.3 Discussion

While the concurrent 4D‐STEM and EDX methodology presented here provides important insights into the nanoscale heterogeneity of LHP thin‐films, care must be taken in the interpretation of results obtained using these complex statistical analyses. Firstly, our NMF‐based analysis assigns a single, dominant crystal structure to each LV. In reality, these nanoscale regions are unlikely to be perfectly structurally pure and may contain complex interfaces, localized strain, or overlapping structures along the beam direction. To mitigate potential misinterpretations, we acquired the datasets from 100 distinct scan regions, covering a total area of 63.5 µm^2^, ensuring our conclusions are based on a statistically robust dataset. Furthermore, while the interpretation of the 2H → 4H → 6H transformation pathway relies on spatial adjacencies, the consistency of these correlations across over 1.6 million individual RVPs provides significant statistical weight to the proposed kinetic sequence. This high‐throughput approach bridges the gap between the high‐resolution detail of TEM and the statistical robustness typically associated with bulk techniques like XRD. Secondly, while our structural identification is strongly supported by correlative EDX data, its low SNR prevents fully quantitative analysis. An improved SNR could be achieved by acquiring datasets from a larger number of scan regions, by increasing the solid angle of the EDX detector, or by performing multiple scans in the same region, which may be possible as we confirmed that our experimental conditions caused minimal damage to the film. However, collecting more data increases both the acquisition time and required computational resources, and as the noise in an EDX measurement is Poissonian, the SNR will increase as the square root of the number of measurements taken, offering diminishing improvements for additional measurements [37, 38].

Despite these limitations, our work represents a significant advance in the nanoscale characterization of beam‐sensitive materials, using LHP films as an exemplar. By combining structural mapping with correlative chemical analysis at the nanoscale, we have provided direct evidence of a complex mosaic of perovskite and non‐perovskite phases coexisting within a state‐of‐the‐art LHP film. While bulk measurements, such as XRD and secondary ion mass spectroscopy (SIMS) by Gratia et al. established a composition‐dependent stabilization threshold (> 3% Cs^+^) [32], our methodology resolves the physical manifestation of this instability at the nanoscale. We show that these hexagonal polytypes are not merely bulk impurities but form a highly fragmented mosaic finely interspersed within the α‐perovskite matrix. The underlying mechanism governing the relationship between Cs deficiency and polytype can be understood by considering the thermodynamics of the Goldschmidt tolerance factor (*t*). As the FA cation is slightly too large to stabilize the α‐phase at room temperature (*t >* 1), the local incorporation of the smaller Cs cation is a thermodynamic requisite for maintaining the photoactive structure [39, 40, 41, 42]. Consequently, we can infer that if a localized nanodomain becomes Cs deficient during crystallization, the local tolerance factor dictates that it must inevitably collapse into the more stable, photoinactive hexagonal polytype.

Furthermore, the spatial correlation revealed in our neighboring phase analysis provides the first direct nanoscale evidence for the 2H → 4H → 6H kinetic transformation pathway. While this sequence has been proposed based on thermodynamic theory and in situ bulk XRD, our ability to map the physical adjacency of these specific polytypes over a statistically robust area (63.5 µm^2^) confirms that this evolution persists even in high‐performance mixed‐ion LHP films. This technique provides a new tool to effectively probe the impact of processing on nanoscale chemical and structural heterogeneity, and its implications on long‐term material stability and device reliability. Beyond halide perovskites, we believe that our methodology will provide crucial nanoscale insights needed to achieve operational stability in a range of multicomponent energy materials.

## 3 Conclusions

In summary, we have developed a low‐dose, concurrent 4D‐STEM and EDX measurement protocol and, coupled with a data‐driven NMF analysis, have addressed the challenge of characterizing beam‐sensitive LHP thin‐films. This approach has enabled the deconvolution of a complex, nanoscale structural and chemical landscape in (FA_0_._83_Cs_0_._17_)Pb(I_0_._8_Br_0_._2_)_3_ thin films that otherwise appear homogeneous in conventional bulk measurements. While our results provide direct evidence of the structural heterogeneity (coexisting crystal structures) within the film, the key scientific novelty of this method is the direct elucidation of the link between chemical composition and crystal structure at the nanoscale. We have shown that the presence of undesirable, photovoltaically inactive, hexagonal polytypes is predominantly caused by significant local deficiencies in cesium. Spatially, these distinct domains are not clustered but are finely interspersed throughout the photoactive α‐perovskite matrix, creating a pervasive network of phase boundaries. The spatial adjacencies of these polytypes also provide evidence for a 2H → 4H → 6H kinetic transformation pathway operating within the film.

The broader implication of this work is that even in state‐of‐the‐art mixed‐ion perovskite compositions engineered for stability, nanoscale chemical inhomogeneity persists and acts as the primary seed for the formation of performance‐limiting phases. Achieving true, long‐term device stability will therefore depend on developing fabrication routes that can ensure compositional homogeneity at the nanoscale. Furthermore, because this analytical strategy relies on unsupervised machine learning to extract crystallographic features from large‐scale SEND datasets, it is inherently material‐agnostic. Therefore, this low‐dose correlative 4D‐STEM‐EDX workflow can be applied to map structure‐property relationships across a wide variety of emerging, beam‐sensitive materials—including investigating how varying global stoichiometry impacts the density and distribution of nanoscale phase impurities in next‐generation photovoltaics or ion batteries. Finally, the next technical phase will involve the implementation of a fully automated acquisition system, complete with an automated process for ROI determination. This development is justified as it is expected to yield statistically more robust results, achieve large area coverage comparable to established bulk techniques (e.g., XRD), and minimize the dependence on manual intervention and extensive operational hours.

## 4 Experimental Section

### 4.1 Sample Preparation

FA_0.83_Cs_0.17_PbI_2.4_Br_0.6_ films were fabricated as follows: 207 mg FAI (Greatcell Solar), 64 mg CsI (Alfa Aesar), 103.8 mg PbBr_2_ (Alfa Aesar), and 538.2 mg PbI_2_ (TCI Chemicals) were dissolved in a 4:1 v/v ratio of DMF:DMSO (Sigma‐Aldrich) to give a 1.3 M perovskite ink. To prepare the TEM specimen, the ink was diluted to 0.5 M using a 4:1 v/v ratio of DMF:DMSO and was spin‐coated directly onto a TEM grid, which is a copper mesh grid with a carbon‐support film, attached to a rigid glass substrate in a glovebox using a two‐step deposition program: 1000 rpm for 10 s, followed by 6000 rpm for 35 s. For the solvent‐quenching step, 300 µL of methyl acetate (Sigma‐Aldrich) was quickly deposited onto the spinning substrate 10 s before the end of the program. The substrates were then directly transferred onto a hotplate and annealed for 45 min at 100°C, after which they were allowed to cool to room temperature naturally. To determine the film thickness, thin‐films were deposited onto glass substrates as detailed above. Using a Dektak surface profileometer, the film thickness was measured in 3 places over 2 identically processed films. The average film thickness was determined to be 80 ± 5 nm.

To ensure that the sample preparation yielded device‐relevant films, films deposited from the more highly concentrated ink (1.3 M) were processed into full p‐i‐n perovskite solar cells. Briefly, prepatterned indium tin oxide (ITO)‐coated glass substrates were sonicated for 15 min each in Decon, deionized water, acetone, and isopropyl alcohol. The substrates then underwent a 30‐min UV–ozone treatment immediately before device fabrication. 200 mL of a self‐assembled monolayer Me4PACz [4‐[(3,6‐dimethyl‐9H‐carbazol‐9‐yl)butyl]phosphonic acid] solution (0.33 mg mL^−1^ in anhydrous ethanol) was then deposited inside an N_2_‐filled glovebox. The solution was deposited onto the substrate and, after waiting for 10 s, spun at 3000 rpm for 20 s with an acceleration of 600 rpm s^−1^. This layer was then annealed for 10 min at 100°C and allowed to cool naturally to room temperature. The perovskite layer was then deposited as described above. After deposition of the perovskite layer, 20 nm C_60_, 5 nm bathocuproine (BCP), and 100 nm Ag were thermally evaporated, and the full devices were left in a N_2_‐filled glovebox overnight prior to device characterization, resulting in the J‐V characteristics illustrated in Figure S1.

### 4.2 X‐Ray Diffraction Measurements

Perovskite samples were deposited onto glass substrates as detailed above. X‐ray diffraction (XRD) spectra were taken using a Panalytical X‐pert powder diffractometer. For collection of the diffractograms, a Cu‐Kα x‐ray source (*λ* = 1.54 Å) was set to 40 kV voltage and 40 mA current.

### 4.3 Concurrent SEND‐EDX Acquisition System

Aberration‐corrected transmission electron microscopy (TEM) was performed on an ARM300CF (JEOL), with a quad‐chip MerlinEM (Quantum Detectors) direct electron detector (DED), and an XMAX100 (Oxford Instruments) energy dispersive x‐ray spectroscopy (EDX) detector. Each instrument is operated via dedicated software installed on individual personal computers (PCs), which are interconnected through an internal local area network. Communication between the various hardware components was achieved through in‐house Python code and transmission control protocol/internet protocol (TCP/IP) communication, with hardware level triggering between the scan generator and detectors. The scheme of the concurrent acquisition is presented in Figure S2, and the detailed procedures are as follows: ① A custom Python program facilitates communication with the DED PC and TEM using TCP/IP and with the EDX PC using representational state transfer application programming interface (REST API); ② the MerlinEM detector is prepared to receive an acquisition trigger from the EDX's scan generator; ③ the EDX software receives a command from the Python script to initiate the EDX acquisition; ④ the scan generator triggers concurrent acquisition on both the DED and EDX detectors; ⑤ The diffraction pattern and EDX spectrum are measured at each scanned pixel and are transmitted to the DED PC and EDX PC, respectively. Note that large‐sized scanning electron nanodiffraction (SEND) data is stored in the data center of Diamond Light Source Ltd. (United Kingdom) through fiber‐optic communication.

Regarding the DED's trigger configuration, the acquisition of each diffraction pattern begins upon the detection of a rising edge signal originating from the EDX's scan generator and stops after the dwell time per pixel (*t*) has elapsed. Within this configuration, the dwell time per pixel for EDX should be larger than *t* due to the extended processing time required by the EDX software; thus, the dwell times per pixel for SEND and EDX were set as *t* ms and 2.0 × *t* ms, respectively. Acquisition parameters, for example, dwell time per pixel and scan shape, can be specified through the graphical user interface (GUI) of the acquisition program, along with the number of times a given area should be scanned.

### 4.4 Data Acquisition and Analysis

The SEND‐EDX data pairs were acquired using an aberration corrected TEM (ARM300CF; JEOL) with an acceleration voltage of 300 kV. Note that the electron probe, a nano‐parallel beam, was formed with the probe‐corrector optics turned off and by changing the probe‐forming lenses in order to minimize the probe convergence semi‐angle. The beam current was 0.5 pA, measured using a Faraday cup, and the probe size was estimated to be 2 nm in diameter—operating under the diffraction limit regime—with a convergence semi‐angle of 0.3 mrad. The total electron beam dwell time per pixel was set to 1.0 ms for constructing the SEND‐EDX dataset (Figure 2 and Figure S3). The EDX integration time was matched to this full 1.0 ms dwell. However, the DED exposure time was set to 0.5 ms (*t*) to avoid pixel saturation, making the EDX acquisition time equal to 2.0 × *t* ms. The ten frames of SEND‐EDX data pairs shown in Figure 1 were individually acquired across four distinct scan regions to monitor beam‐induced damage. Translational scan drift was corrected for each dataset by calculating the phase correlation shifts between frames. The number of the SEND‐EDX data pairs used in the correlative characterization of the LHP sample was 100 (*N*). The electron beam was blanked when moving the sample to ensure that no damage to the sample had occurred before measurements were taken. The scan shape, *S_x_* × *S_y_* was 128 × 127 pixels with a scan step of 6.25 nm, and each diffraction pattern comprises 515 × 515 (*S_dx_* × *S_dy_*) pixels with a pixel size of 0.0091 × 0.0091 1/Å^2^. The step size was chosen as an optimal sampling strategy; it is sufficiently large to prevent spatial overlap of the core electron beam (thereby minimizing cumulative beam damage), yet fine enough to capture the localized, distinct phase boundaries. Using a gold cross grating reference sample, scan distortions and the ellipticity of the diffraction patterns were corrected, and pixel sizes were also accurately calculated. Each EDX spectrum has 2048 energy channels (*S_e_*) with an energy step of 0.01 keV/channel. The EDX collection solid angle is less than 0.5 sr.

Each diffraction pattern was transformed into an RMP and RVP after aligning its center beam position using the polar transform method of the py4D‐STEM Python package (Figure S3) [43]. The radially flattened profiles have 365 reciprocal distance steps (*S_r_*), and were merged into a data matrix by flattening *N* × *S_x_* × *S_y_*; consequently, its dimensions were *N* × *S_x_* × *S_y_* × *S_r_*. Before applying NMF to the data matrix [30, 44], the reciprocal distance (*k*) was limited in the range of 0.2–0.6 1/Å as the effective analysis range, where high variance values mostly appeared. Consequently, the final data matrix comprised 1 625 600 RVPs × 44 reciprocal distance steps. NMF was performed to decompose the data matrix, and the number of loading vectors (LV) was chosen as 9 (*N_lv_*); in other words, each 3D radial variance profile data, *S_x_* × *S_y_* × *S_r_* was reduced into the *N_lv_* coefficient maps, *S_x_* × *S_y_* × *N_lv_*. To extract the class‐averaged RVPs and EDX spectra, high‐coefficient masks were generated using a top 10% coefficient threshold. This value was specifically chosen to ensure the spatial purity of the structural domains while maintaining sufficient aggregate x‐ray counts for reliable quantification (Figure S14). Subsequently, distinct clustered regions in each high‐coefficient map were screened using a clustering algorithm—specifically, density‐based spatial clustering of applications with noise (DBSCAN)—and a concave hull method [24, 25]. The averaged radially flattened profiles and the averaged EDX spectra were calculated only for these clustered areas. The powder x‐ray diffraction patterns of the candidate phases were simulated using the py4D‐STEM and pymatgen Python packages as shown in Figure S12 [43, 45]. Note that the simulated XRD patterns were Gaussian‐blurred to make the full‐width‐at‐half‐maximum (FWHM) of their peaks comparable to that of nanobeam electron diffraction (NBED) patterns. Furthermore, the method for phase identification depends on the comparison of peak positions; hence, a phase with a relatively high frequency of Bragg reflections, such as γ‐CsPbI_3_, will dominate the best match results without the blurring process.

The peak positions of both the class‐averaged RVPs and the simulated XRD patterns were extracted using the “*find_peaks*” method from the SciPy Python package [46]. To reliably identify a crystal phase, matching at least three peak positions is generally considered necessary. Accordingly, three prominent peaks were extracted from the class‐averaged RVPs for all LVs, with the exception of LV8, which yielded only two distinct peaks. To quantitatively assign material phases, a peak‐position matching score was calculated for each LV (see Table S3 and Supplementary Text for details). The centroids of each cluster and the distances between them were calculated to obtain information about the nearest LV for each LV. After collecting these distances, the percentages of the three closest neighboring LVs for each source LV were calculated. EDX quantification was implemented using the eXSpy package [47].

## Author Contributions

**Jinseok Ryu and Christopher S. Allen**: conceptualization. **Jinseok Ryu, Mohsen Danaie, David G. Hopkinson, Katherine E. MacArthur, and Christopher S. Allen**: methodology. **Jinseok Ryu, Mohsen Danaie, David G. Hopkinson, Katherine E. MacArthur, and Christopher S. Allen**: software. **Jinseok Ryu, Alexandra A. Sheader, and Nakita K. Noel**: investigation. **Jinseok Ryu**: visualization. **Christopher S. Allen and Nakita K. Noel**: funding acquisition. **Christopher S. Allen and Nakita K. Noel**: supervision. **Jinseok Ryu**: writing – original draft. **Mohsen Danaie, Nakita K. Noel, and Christopher S. Allen**: Writing – review and editing.

## Conflicts of Interest

The authors declare no conflicts of interest.

## Supporting information




**Supporting File**: adfm74382‐sup‐0001‐SuppMat.docx.

## Data Availability

All codes of the data analysis pipeline are publicly available in the following link: https://github.com/ePSIC‐DLS. The Zenodo link to the raw datasets will be included once the article is accepted and obtains https://doi.org/10.5281/zenodo.21514231.
