# Supplementary material for: Resolving Nanoscale Heterogeneities in Lead Halide Perovskites Through Low‐Dose Concurrent 4D‐STEM‐EDX Mapping
Source: Adv Mater. 2026 Jul 31;38(55):e74382. doi: 10.1002/adma.74382 (PMC13629251; doi:10.1002/adma.74382)
Supplement: Supplementary file 1 [file ADMA-38-e74382-s001.docx]

Supporting Information

**Resolving nanoscale heterogeneities in lead halide perovskites through low dose concurrent 4DSTEM-EDX mapping**

Jinseok Ryu^1^, Alexandra A. Sheader^2^, Mohsen Danaie^1^, David G. Hopkinson^1^, Katherine E. MacArthur^3^, Nakita K. Noel^2*^ and Christopher S. Allen^1,4*^

**Affiliations:**

^1^electron Physical Science Imaging Centre, Diamond Light Source Ltd.; Didcot, OX11 0DE, United Kingdom.

^2^Department of Physics, University of Oxford; Oxford, OX1 3PU, United Kingdom.

^3^Oxford Instruments Materials Analysis; High Wycombe, HP12 3SE, United Kingdom.

^4^Department of Materials, University of Oxford; Oxford, OX1 3PH, United Kingdom.

*Corresponding authors. Email: christopher.allen@diamond.ac.uk, nakita.noel@physics.ox.ac.uk

Table of Contents

**Supplementary Text.** Pseudocode for calculating the matching scores

**Fig. S1.** Photovoltaic performance of a representative FA_0.83_Cs_0.17_Pb(I_0.8_Br_0.2_)_3_ solar cell.

**Fig. S2.** Scheme for constructing numerous SEND-EDX data pairs.

**Fig. S3.** Sample stage locations of each scan region of the datasets.

**Fig. S4.** Virtual annular dark-field images of each scan region

**Fig. S5.** Determination of the optimal number of loading vectors (LVs).

**Fig. S6.** Coefficient maps of all scan regions.

**Fig. S7.** High coefficient masks of all scan regions.

**Fig. S8.** Class-averaged energy dispersive X-ray (EDX) spectra.

**Fig. S9.** Number of EDX spectra vs. counts per spectrum.

**Fig. S10.** X-ray diffraction pattern of an as-prepared FA_0.83_Cs_0.17_Pb(I_0.8_Br_0.2_)_3_ film.

**Fig. S11.** X-ray diffraction pattern simulation result.

**Fig. S12.** Averaged original diffraction patterns of each LV1 clustered region.

**Fig. S13.** Effect of coefficient thresholding on high-coefficient mask generation.

**Fig. S14.** Impact of thresholding parameters on the spatial purity of the structural domains.

**Table S1.** Total number of binned pixels and the corresponding integrated X-ray counts for each class-averaged energy dispersive X-ray (EDX) spectrum.

**Table S2.** Candidate phases and their crystal systems used in X-ray diffraction simulation.

**Table S3.** Peak positions in class-averaged radial variance profiles (RVPs) of loading vectors (LVs).

**Table S4.** Total peak-position matching result.

**Table S5.** Percentages of the three closest neighbouring loading vectors (LVs) for each source LV.

Supplementary Text

Pseudocode for calculating the matching scores

**Algorithm:** Calculate the peak-position matching score of a loading vector (LV) and phase

**Input:**

- XRD_Peaks: A list of numerical values representing the peak positions of a powder X-ray diffraction (XRD) pattern for a specific phase.
- LV_Peaks: A list of numerical values representing the peak positions of a class-averaged radial variance profile (RVP).

**Output:**

- Matching_Score: A single numerical value representing the cumulative matching score.

1. Initialize Matching_Score to 0.
2. **For** each Peak_LV in LV_Peaks:
   1. Initialize Min_Difference to infinity.
   2. **For** each Peak_XRD in XRD_Peaks:
      i. Calculate the absolute difference:

Difference = |Peak_XRD - Peak_LV|.

ii. **If** Difference < Min_Difference:

Update Min_Difference = Difference.

1. Add Min_Difference to the total score:
   i. Matching_Score = Matching_Score + Min_Difference.

3. **Return** Matching_Score.

Fig. S1. Photovoltaic performance of a representative FA_0.83_Cs_0.17_Pb(I_0.8_Br_0.2_)_3_ solar cell. Current density–voltage (J-V) characteristics of a representative FA_0.83_Cs_0.17_Pb(I_0.8_Br_0.2_)_3_ solar cell, with a power conversion efficiency (η) of 20.5%, short-circuit current density ($\boldsymbol{J}_{\boldsymbol{sc}}$) of 20.6 mA cm⁻², an open-circuit voltage ($\boldsymbol{V}_{\boldsymbol{oc}}$) of 1.2 V, and FF of 83%, validating the baseline quality of the thin -ilm. To ensure direct comparability, the device was fabricated from the same precursor ink (before dilution) and in parallel with the specimens deposited on TEM grids and analysed in the main text.

Fig. S2. Scheme of the concurrent 4DSTEM-EDX acquisition workflow. Colours indicate individual manufacturers.

Fig. S3. Scheme for constructing numerous SEND-EDX data pairs. The scan shape of each data pair is [*S_x_*×*S_y_*], and the shape of diffraction patterns is [*S_dx_*×*S_dy_*]. The numbers of energy channels and reciprocal distances are *S*_e_ and *S_r_*, respectively.

Fig. S4. Sample stage locations of each scan region of the datasets. The digits indicate the acquisition order. The median distance between adjacent locations is 9.6 μm.

Fig. S5. Virtual annular dark-field images of each scan region. The intensities were collected for the reciprocal distance range of 0.2-0.6 Å^-1^. The digits indicate the acquisition order of each row. Scale bar represents 100 nm.

Fig. S6. Determination of the optimal number of loading vectors (LVs). Errors between the original and the reconstructed data matrices from non-negative matrix factorization (NMF) and their differences with an increasing number of LVs. The largest decrease in error occurs at nine LVs before the first differentiation begins to plateau; therefore, nine LVs are considered sufficient to represent the features of the radial variance profile (RVP) dataset.

Fig. S7. Coefficient maps of all scan regions. Scale bar represents 100 nm.

Fig. S8. High coefficient masks of all scan regions. Scale bar represents 100 nm.

Fig. S9. Class-averaged energy dispersive X-ray (EDX) spectra. Class-averaged EDX obtained by selectively collecting EDX spectra using the high coefficient masks of each loading vector (LV).

Fig. S10. Number of EDX spectra vs. counts per spectrum. Note that the number of spectra is plotted on a logarithmic scale. The inset graphs are examples of EDX spectra with different total counts.

Fig. S11. X-ray diffraction pattern of an as-prepared FA_0.83_Cs_0.17_Pb(I_0.8_Br_0.2_)_3_ film.

Fig. S12. X-ray diffraction pattern simulation result.

Fig. S13. Averaged original diffraction patterns of each LV1 clustered region. Scale bar represents 0.5 Å^-1^.

Fig. S14. Effect of coefficient thresholding on high-coefficient mask generation. The first column displays the high-coefficient masks of all loading vectors (LVs) overlaid on the virtual bright-field image. The second column presents only the high-coefficient masks to emphasise overlaps between distinct clustered regions of the LVs. The final column illustrates the high-coefficient masks specifically for LV6. Instances of undesirable overlap are highlighted with red-bordered boxes in the panels of the second and third columns. Scale bars = 100 nm.

| **Loading Vector** | **Total Number of Binned Pixels** | **X-ray Counts for the Entire Energy Range** | **Net Pb M-Edge Counts**  **(2.2-2.6 keV)** | **Estimated Relative Statistical Error** |
| --- | --- | --- | --- | --- |
| LV1 | 59,494 | 27,229 | 18 | 24 % |
| LV2 | 118,718 | 90,155 | 73 | 12 % |
| LV3 | 56,795 | 38,184 | 29 | 19 % |
| LV4 | 149,566 | 99,714 | 82 | 11 % |
| LV5 | 143,186 | 148,725 | 112 | 9 % |
| LV6 | 117,282 | 108,936 | 83 | 11 % |
| LV7 | 146,407 | 117,252 | 87 | 11 % |
| LV8 | 104,649 | 90,958 | 83 | 11 % |
| LV9 | 74,685 | 72,100 | 50 | 14 % |

Table S1. Total number of binned pixels, the corresponding integrated X-ray counts and estimated relative Poisson statistical errors for each class-averaged EDX spectrum.

| **Phase Name** | **Crystal System** | **Reference** |
| --- | --- | --- |
| 2H-PbI_2_ | Hexagonal | (*1*) |
| 3R-PbI_2_ | Rhombohedral | (*1*) |
| γ-CsPbI_3_ | Orthorhombic | (*2*) |
| α-FAPbBr_3_ | Cubic | (*3*) |
| α-FAPbI_3_ | Cubic | (*4*) |
| β-FAPbI_3_ | Tetragonal | (*4*) |
| 2H | Hexagonal | (*4*) |
| 4H | Hexagonal | (*5*) |
| 6H | Hexagonal | (*5*) |

Table S2. Candidate phases and their crystal systems used in X-ray diffraction simulation.

| **LV1** | **LV2** | **LV3** | **LV4** | **LV5** | **LV6** | **LV7** | **LV8** | **LV9** |
| --- | --- | --- | --- | --- | --- | --- | --- | --- |
| 0.218 | 0.272 | 0.299 | 0.317 | 0.272 | 0.308 | 0.218 | 0.308 | 0.272 |
| 0.308 | 0.472 | 0.472 | 0.472 | 0.354 | 0.381 | 0.308 | 0.472 | 0.345 |
| 0.444 | 0.544 | 0.571 | 0.544 | 0.472 | 0.463 | 0.444 |  | 0.535 |

Table S3. Peak positions in class-averaged radial variance profiles (RVPs) of loading vectors (LVs). Unit: Å^-1^

|  | **α-FAPbI_3_** | **β-FAPbI_3_** | | **2H** | | **4H** | | **6H** | | **2H-PbI_2_** | | **3R-PbI_2_** | | **γ-CsPbI_3_** | | **α-FAPbBr_3_** | |
| --- | --- | --- | --- | --- | --- | --- | --- | --- | --- | --- | --- | --- | --- | --- | --- | --- | --- |
| **LV1** | **0.012** | | 0.024 | | 0.019 | | 0.034 | | 0.018 | | 0.096 | | 0.055 | | 0.020 | | 0.065 |
| **LV2** | **0.002** | | 0.016 | | 0.018 | | 0.008 | | 0.018 | | 0.046 | | 0.017 | | 0.012 | | 0.026 |
| **LV3** | 0.021 | | 0.025 | | 0.014 | | **0.012** | | 0.016 | | 0.020 | | 0.027 | | 0.014 | | 0.017 |
| **LV4** | **0.005** | | 0.013 | | 0.014 | | 0.016 | | 0.015 | | 0.054 | | 0.016 | | 0.013 | | 0.025 |
| **LV5** | **0.004** | | 0.012 | | 0.016 | | 0.011 | | 0.013 | | 0.058 | | 0.020 | | 0.015 | | 0.036 |
| **LV6** | 0.020 | | 0.032 | | **0.009** | | 0.034 | | 0.018 | | 0.020 | | 0.045 | | 0.029 | | 0.037 |
| **LV7** | **0.012** | | 0.024 | | 0.019 | | 0.034 | | 0.018 | | 0.096 | | 0.055 | | 0.020 | | 0.065 |
| **LV8** | 0.006 | | 0.015 | | **0.003** | | 0.017 | | 0.014 | | 0.027 | | 0.022 | | 0.011 | | 0.020 |
| **LV9** | 0.017 | | 0.018 | | 0.019 | | 0.011 | | **0.006** | | 0.066 | | 0.011 | | 0.021 | | 0.036 |

Table S4. Total peak-position matching result. The bold values correspond to the lowest scores in each row. Unit: Å^-1^

|  | **Neighbouring LV** |
| --- | --- |

|  | | **LV1**  **(a-Carbon)** | **LV2**  **(α-FAPbI_3_)** | **LV3**  **(4H)** | **LV4**  **(α-FAPbI_3_)** | **LV5**  **(α-FAPbI_3_)** | **LV6**  **(2H)** | **LV7**  **(α-FAPbI_3_)** | **LV8**  **(2H)** | **LV9**  **(6H)** |
| --- | --- | --- | --- | --- | --- | --- | --- | --- | --- | --- |
| **Source LV** | **LV1**  **(a-Carbon)** | **15.3** | 10.6 | 11.7 | 10.3 | 10.1 | 8.7 | 14.1 | 9.9 | 9.3 |
|  | **LV2**  **(α-FAPbI_3_)** | 3.1 | 9.7 | 13.7 | 13.8 | 12.8 | 7.2 | 14.3 | **15.2** | 10.1 |
|  | **LV3**  **(4H)** | 3.6 | 14 | 6.7 | 12.5 | 14.6 | 6.7 | 14.3 | **16.6** | 11 |
|  | **LV4**  **(α-FAPbI_3_)** | 3.4 | 14.2 | 11.6 | 7.9 | **18.5** | 6.9 | 14.8 | 15.2 | 7.5 |
|  | **LV5**  **(α-FAPbI_3_)** | 2.8 | 12 | 14 | **18** | 9.1 | 6.7 | 14 | 13.9 | 9.6 |
|  | **LV6**  **(2H)** | 4.6 | **14.6** | 12.3 | 13.2 | 12.7 | 8 | 12.3 | 12.5 | 9.8 |
|  | **LV7**  **(α-FAPbI_3_)** | 4.7 | 14 | 12.8 | 15.4 | 13.7 | 6 | 8.1 | **16.3** | 8.9 |
|  | **LV8**  **(2H)** | 2.9 | 15.2 | 15.1 | 13.9 | 13.4 | 6.4 | **15.8** | 8.2 | 9.3 |
|  | **LV9**  **(6H)** | 4.7 | 13.6 | **15.3** | 10.7 | 13 | 7.7 | 12.4 | 13.7 | 8.9 |

Table S5. Percentages of the three closest neighbouring loading vectors (LVs) for each source LV. The bold values correspond to the highest percentage in each row. Each row has a diverging color scale: the higher the value, the redder the cell; the lower the value, the bluer the cell. Unit: %

References

1.	R. Wyckoff, Interscience publishers, new york, new york rocksalt structure. *Crystal structures* **1**, 85-237 (1963).

2.	D. B. Straus, S. Guo, R. J. Cava, Kinetically stable single crystals of perovskite-phase CsPbI3. *Journal of the American Chemical Society* **141**, 11435-11439 (2019).

3.	C. Li, E. J. Juarez-Perez, A. Mayoral, Atomic-level understanding of a formamidinium hybrid halide perovskite, FAPbBr 3. *Chemical Communications* **58**, 12164-12167 (2022).

4.	J. Yin, G. Teobaldi, L.-M. Liu, The role of thermal fluctuations and vibrational entropy: A theoretical insight into the δ-to-α transition of FAPbI3. *The Journal of Physical Chemistry Letters* **13**, 3089-3095 (2022).

5.	P. Gratia *et al.*, The Many Faces of Mixed Ion Perovskites: Unraveling and Understanding the Crystallization Process. *ACS Energy Letters* **2**, 2686-2693 (2017).
